# Microbe Profile: *Bacillus subtilis*: model organism for cellular development, and industrial workhorse

**DOI:** 10.1099/mic.0.000922

**Published:** 2020-05-11

**Authors:** Jeffery Errington, Lizah T van der Aart

**Affiliations:** ^1^​ Centre for Bacterial Cell Biology, Biosciences Institute, Newcastle University, Newcastle-upon-Tyne, NE2 4AX, UK

**Keywords:** *Bacillus subtilis*, biofilm, endospore, protein secretion, plant protection, transformation

## Abstract

*
Bacillus subtilis* is the best studied model organism of the Gram-positive lineage. It is naturally transformable and has an extremely powerful genetic toolbox. It is fast growing and easy to cultivate. It is an important industrial organism, being proficient at secreting proteins and making small fine chemicals, as well as acting as a plant growth promoter. It has been an important model system for studying biofilms. Finally, it makes endospores, which have provided an exceptionally fruitful system for studying various central problems of cellular development, including the generation of asymmetry, cell fate determination and morphogenesis.

## Taxonomy

Domain *Bacteria*, phylum *Firmicutes *, class *Bacilli *, order *Bacillales *, family *Bacillaceae *, genus *Bacillus *, species *subtilis*.

## Properties

*B. subtilis* is a fast-growing, Gram-positive, aerobic bacterium with rod-shaped cells that are typically 2–6 µm long and just less than 1 µm in diameter. The optimal growth temperature is about 30–35 C, giving a doubling time of as little as 20 min. Under some growth conditions the cells have a tendency to form long chains connected by uncleaved septal wall material. Under starvation conditions the cells can undergo a complex-2 cell-differentiation process leading to the formation of an endospore, which is released by lysis of the enveloping mother cell. The vegetative cells can be motile. Alternatively, they can form biofilms and ‘fruiting bodies’ containing spores.

## Genome

The widely studied *B. subtilis* strain 168 is a tryptophan auxotroph isolated in the 1950s. Its was one of the first bacteria to be fully genome sequenced, revealing a 4.2 Mbp chromosome with about 4100 genes [1]. The genome of *B. subtilis* remains one of the best annotated, through a series of updates, most recently that of [2]. A comprehensive database, ‘SubtiWiki’ (http://subtiwiki.uni-goettingen.de/), provides a reliable and user-friendly interface to the latest data. The database includes a hugely comprehensive set of data listing transcriptional units, promoters and regulatory RNAs from the work of Nicolas *et al*. [3]. Complete lists of essential genes have been obtained in a series of global projects, most recently identifying 257 genes required for growth in LB at 37°C [4]. Analysis of the complete genome sequences of 36 diverse *B. subtilis* isolates has revealed a ‘pan genome’ (total gene set) of about 6250 genes, and a ‘core genome’ (conserved gene set) of about 2500 genes [2]. Notable gene classes include about 300 genes required for endospore formation, and multiple prophages or phage remnants. Conclusions from an overview of gene content are consistent with the notion that *B. subtilis* is adapted for life on plants or in the rhizosphere.

## Phylogeny

*B. subtilis* is the ‘type strain’ of the order *Bacillales* and the defining organism of the whole *Firmicutes* phylum, having been first described in detail by Ferdinand Cohn in 1872 [5]. Cohn’s organism was probably identical to an organism isolated even earlier, in 1832, by Ehrenberg. The history of the discovery and characterization of *B. subtilis *, and controversies over its taxonomic status, are summarized in an interesting article by Soule [6].

The most recent version of Bergey’s Manual lists 141 species of *Bacillus* [7]. A wide range of traits are used to distinguish between *B. subtilis* and other species in the Genus. Most prominent among these are types of murein (peptidoglycan) cross bridging; ability to hydrolyse and utilize various carbon sources; colony, cell and spore morphology; and tolerance of salt, pH and temperature variation.

## Key features and discoveries

*B. subtilis* has a long history, being first described in the nineteenth century. The origins of the standard lab strain, 168, are poorly documented, but its place in the annals of genetics was cemented by experiments in the late 1950s showing that it was naturally transformable with linear DNA (see [8]). *B. subtilis* emerged as the Gram-positive model organism of choice largely because endospore formation became popular as a marvellously tractable system for studying fundamental aspects of cellular development and differentiation. Processes such as the decision to initiate sporulation, asymmetric cell division, cell fate determination and cell morphogenesis were all worked out in molecular detail at a time when it was very difficult to dissect these processes in higher organisms.

A pivotal problem in understanding spore development lay in discriminating between events occurring simultaneously in the developing prespore and mother-cell compartments, which have identical chromosomes but very different gene expression profiles. This problem powered the adaptation of digital fluorescence imaging for use in bacteria, which was then a major factor ushering in the modern field of bacterial cell biology. Later, these methods were applied to many other important problems, especially central bacterial cell processes of cell division, chromosome segregation, and cell growth and morphogenesis. Progress in understanding these processes now runs almost in parallel between *B. subtilis* and its Gram-negative comparator, *Escherichia coli. Bacillus* genetics and cell-biology methods have also made the organism popular for more general studies of cell physiology and biochemistry, as well as alternative morphogenic processes, such as biofilm formation.

Another major driver of interest in *B. subtilis* is based on its importance as an industrial organism, mainly through its prodigious ability to secrete various important hydrolytic enzymes directly into the culture medium but also as a producer of fine chemicals, such as riboflavin. Its attractiveness as a safe host for production of natural and engineered products has been helped by its long standing use in ‘natto’, a Japanese dish made from fermented soy bean curd and also as a probiotic. As mentioned above, *B. subtilis* appears to be adapted to life in association with plants, either as an epiphyte or in the rhizosphere, and historically it has typically been isolated from decaying vegetative matter such as hay. Adaptation to this ecological niche may help explain a third important industrial use of *B. subtilis *, as a plant growth promoter, through production of specialized metabolites, niche exclusion of pathogens and other probably various other factors.

## Open questions

*B. subtilis* and the wider *Firmicute* lineage are usually referred to as Gram-positive, but some *Firmicutes* have a genuine outer membrane. What is the explanation for this profound evolutionary conundrum?

Can we use *B. subtilis* to solve the fundamental mechanistic questions surrounding cell morphogenesis and cell division?

*B. subtilis* is a wonderful host for production of hydrolytic enzymes from other bacteria but can the secretion mechanism be made to work for heterologous, high-value proteins?

What ecological factors and molecular mechanisms underlie the plant growth promoting properties of *B. subtilis *?

## References

[1] Kunst F, Ogasawara N, Moszer I, Albertini AM, Alloni G (1997). The complete genome sequence of the Gram-positive bacterium Bacillus subtilis. Nature.

[2] Borriss R, Danchin A, Harwood CR, Médigue C, Rocha EPC (2018). *Bacillus subtilis,* the model Gram-positive bacterium: 20 years of annotation refinement. Microb Biotechnol.

[3] Nicolas P, Mäder U, Dervyn E, Rochat T, Leduc A (2012). Condition-dependent transcriptome reveals high-level regulatory architecture in Bacillus subtilis. Science.

[4] Koo B-M, Kritikos G, Farelli JD, Todor H, Tong K (2017). Construction and analysis of two genome-scale deletion libraries for *Bacillus subtilis*. Cell Syst.

[5] Cohn F (1872). Untersuchungen über Bacterien. Beitrage zur Biologie der Pflanzen Heft 2.

[6] Soule MH (1932). Identity of *Bacillus subtilis*, Cohn 1872. J Infect Dis.

[7] Logan NA, de Vos P (2009). Genus I. *Bacillus* Cohn 1872. Bergey’s Manual of Systematic Bacteriology Second Edition, Volume 3, The Firmicutes.

[8] Zeigler DR (2011). The genome sequence of *Bacillus subtilis* subsp. spizizenii W23: insights into speciation within the *B. subtilis* complex and into the history of *B. subtilis* genetics. Microbiology.

